# Effective method for upcycling construction and demolition waste into concrete: A life cycle approach

**DOI:** 10.1177/0734242X231180651

**Published:** 2023-06-24

**Authors:** Abhijit Mistri, Cheela Venkata Ravi Sankar, Brajesh Kumar Dubey, Navdeep Dhami, Sriman Kumar Bhattacharyya, Abhijit Mukherjee, Sudhirkumar V Barai

**Affiliations:** 1 Department of Civil Engineering, Indian Institute of Technology, Kharagpur, West Bengal, India; 2 School of Civil and Mechanical Engineering, Curtin University, Bentley, WA, Australia; 3 Fibre and Particle Engineering Research Unit, Faculty of Technology, University of Oulu, Oulu, Finland; 4 Environmental Engineering and Management, Department of Civil Engineering, Indian Institute of Technology Kharagpur, Kharagpur, West Bengal, India; 5 Department of Civil Engineering, MVGR College of Engineering (Autonomous), Vizianagaram, Andhra Pradesh, India; 6 Department of Civil Engineering, Birla Institute of Technology & Science, Pilani, Rajasthan, India

**Keywords:** Construction and demolition waste, recycled coarse aggregate, life cycle analysis, treatment methods, waste management

## Abstract

Different property enhancement techniques have already been established to support upcycling of construction and demolition waste as aggregate in concrete. However, the most suitable and sustainable method is still unknown. Quality improvement of recycled coarse aggregate (RCA) after any treatment method and its environmental impact is estimated using life cycle analysis (LCA). This article compares the environmental impacts of such treatment methods on RCA and aims to find out the most suitable method with minimum impacts. The functional unit of this study is considered the preparation of 1 tonne of treated aggregate (recycled), considering reduction in water absorption after the treatment. An LCA is carried out using the SimaPro software (https://simapro.com/) followed by ISO 14040/44 guidelines. Based on the LCA environmental profiles, thermal treatment is the highest emission contributing removal method followed by mechanical grinding. In strengthening of attached mortar methods, accelerated carbonation process is the major emission contributing method followed by a specific microbial treatment. Moreover, a sensitivity analysis was performed by varying the energy mix with a focus on renewable-based energy mix. The sensitivity analysis shows a shift on selection for the suitable treatment method and other possibilities considering renewable-based energy mix. A preliminary assessment and probable impact prediction could be conceptualized before the adoption of any treatment method on RCA for a particular location.

## Introduction

Waste from different industries has become a serious problem for many countries as they need special attention to dispose of (Utegenova et al., 2020; Zhang et al., 2020). The generation of municipal solid waste (MSW) has been growing rapidly worldwide (Coelho and de Brito, 2012; Fan et al., 2020) and wastes generated from the construction industry contribute about 40% to the total MSW (Kisku et al., 2017; Mistri et al., 2020; Tam, 2008). This huge waste from the concrete industry and other associated projects builds environmental issues (Behera et al., 2014; Martínez-Lage et al., 2020). Moreover, the scarcity of raw materials and the huge demand for construction aggregates lead our focus towards sustainable practices (Behera et al., 2014; Evangelista and de Brito, 2007). Environmental sustainability has grown to be one of the important aspects of construction projects (Caldas et al., 2021). Therefore, an effective disposal method on construction and demolition (C&D) waste is imperative with an aim for green construction (Tam et al., 2018; Xiao, 2018).

The construction industry itself is responsible for about 7% of the global CO_2_ emissions (Hanifa et al., 2023; Hossain et al., 2016; Statista, 2023; UNEP, 2018). Moreover, the emission from the construction sector is projected to be augmented by about 10% by 2060 due to increase in construction activities (UNEP, 2018). Therefore, research on sustainable construction materials and minimization of greenhouse gas (GHG) emissions are the leading focuses in the concrete industry (Xiao, 2018). In 2020, GHG emissions per capita in European Union (EU) fallen almost 40% compared to that of 1990 (Mølgaard, 1995; Pacheco-Torgal et al., 2016). Additionally, the EU has also planned to reduce it by 80–90% by 2050 compared to the same level in 1990 (European Commission, 2015). One possible and effective way to combat this emission could be substitution of energy-intensive virgin materials with C&D waste without sacrificing material performance (Mehta and Monteiro, 2015; Peris Mora, 2007). In concrete, aggregate occupies about 75–80% of the total volume (Alexander and Mindess, 2005; Mehta and Monteiro, 2015) and the demand has been growing day by day. The use of C&D waste as aggregate avoids the utilization of virgin aggregate in concrete. This conserves natural resources and also equips in handling and management of C&D waste. Aggregate that comes from crushing of C&D waste is known as recycled aggregate (RA) (Mamirov et al., 2022; Mistri, 2022; Tam, 2008). The quality of RA is highly dependent on the source of the old parent concrete (Kou and Poon, 2015; Pedro et al., 2014) and its physiochemical characteristics (Xiao, 2018).

The primary problem associated with the use of recycled coarse aggregate (RCA) in fresh concrete is the existence of attached mortar (AM) on the aggregate surface which is porous in nature (Mistri et al., 2023; Mukharjee and Barai, 2015; Ouyang et al., 2023). Because of the high porosity of AM, water absorption increases in RCA leading to inferior performance of concrete. Additionally, weak interfacial transition zones of RCA result in the poor structural performance of concrete (Behera et al., 2014; Li et al., 2012). It is also reported that the compressive strength of recycled aggregate concrete (RAC) with 100% replacement of natural aggregate concrete (NAC) by RCA shows 30% lesser strength than NAC (Pradhan et al., 2017; Xiao, 2018). Because of high water absorption and associated issues, the utilization of RCA in concrete is not stimulating.

Different property enhancement methods have been studied by researchers (Mistri et al., 2020; Shi et al., 2016; Xiao, 2018) to overcome the drawbacks of RCA for its effective use in concrete. In most cases, the intended objective is to reduce water absorption and improve overall aggregate properties (both the physical and mechanical performance). Primarily, there are mainly two types of treatment: (1) removal of AM (like pre-soaking into water, mechanical grinding, chemical treatment, thermal treatment, etc.) and (2) strengthening of AM (emulsion polymer, carbonation, bio-deposition of calcium carbonate, cement slurry coating, etc.) (Mistri et al., 2020; Pan et al., 2017; Shi et al., 2016). A systematic figure to show the improvement of RCA properties after treatment is given in Supplemental Figure S1. Modification in mixing methods and the use of pozzolanic materials and nanomaterials are also studied by several researchers to improve RAC properties (Li et al., 2009). Recent research found that RCA can be strengthened using a cost-effective and environmentally friendly approach without removing the AM (Mistri et al., 2022; Wang et al., 2017). Research on the strengthening of AM is an active area of interest. However, the most suitable and sustainable method warrants further investigation.

To combat this problem, a better understanding of different available methods on RCA, their advantages/disadvantages, and most importantly the assessment of environmental impacts is crucial. Moreover, before adopting any property enhancement method on RCA, the very basic question arises: (1) is the chosen method capable of improving its drawbacks (primarily aggregate properties, like water absorption), and if so then (2) does the method support sustainability in the concrete industry with minimum environmental impacts? Although there are different methods available in the literature for RCA, however, the detailed life cycle analysis (LCA) on different treatment methods has rarely investigated. Therefore, for the first time, this study is conducted to identify the most suitable method to address the global C&D waste disposal problem from the environmental point of view. An LCA on the existing treatment methods for RCA has been performed. The most common methods that have potential to improve quality (i.e. water absorption) of RCA were selected for LCA. The aim is to determine environmental profiles of different removal and strengthening methods that are available in the literature to identify the method with minimum environmental impacts. Moreover, a sensitivity analysis has also been performed by varying the energy mix with a focus on renewable-based energy in future. The results will provide a useful reference to the research community, practising engineers and policymakers to think insight about the global C&D waste disposal problem and possible mitigation solutions based on LCA of a particular location.

## Different treatment methods on RCA

Existing methods on RCA with the aim for enhancement of properties are available in the literature (Mistri et al., 2020; Shi et al., 2016). This section describes additional information related to the processes for different treatment methods that are available. The same description of the process will be used in the LCA inventory of a particular method. Some of the treatment methods on RCA with the specific procedure are given below:

### Autogenous cleaning process

A rotating drum mill of 30 cm diameter and 50 cm height is the primary equipment for autogenous cleaning (Pepe, 2015). About 33% of the total capacity is filled, and during the rotating operation, a speed of 60 rpm is maintained for 15 minutes. After the process of rotating, the aggregates are washed with water to remove loose particle and dust.

### Mechanical grinding

A modified concrete mixer of a capacity of 8 m^3^ is used for the treatment (Dimitriou et al., 2018). Field sourced RCA is placed inside the mixer and operated with a rotation speed of 10 rpm for 5 hours. Water is added during the process of rotation for better efficiency.

### Thermal treatment

In this treatment, RCA is first kept inside an electric furnace at 500°C for 2 hours (de Juan and Gutiérrez, 2009). After that, the RCA is immediately immersed in cold water. This sudden thermal shock on aggregate helps to remove AM from parent rock by creating stress due to temperature gradient.

### Acid treatment

Acid treatment on RCA is conducted at room temperature using a flux (Tam et al., 2007). Untreated RCA is soaked in different acid solutions (HCl, H_2_SO_4_, H_3_PO_4_) with a molarity of 0.1M. After the treatment for 24 hours, the aggregates are washed with fresh water to remove harmful ions from acid.

### Polymer impregnation

In this treatment, first, dry RCAs are placed inside a desiccator (Kou and Poon, 2010). A vacuum pump is attached to it and the operating pressure is 920 mbar for 6 hours. Polyvinyl alcohol (PVA) solution (6–12%) is added separately to the desiccator. Moreover, 10% PVA solution is recommended as reported. The aggregates are soaked with the solution inside the desiccator for 24 hours with the pump turned on. After that, the aggregates are removed from the chamber and tested.

### Bio-deposition of calcium carbonate

Bio-deposition of calcium carbonate, commonly known as microbial-induced calcium carbonate precipitation (MICP) treatment on RCA, is involved with several steps. First, the bacterial culture media is prepared using specified chemicals and deionized water. Bacteria is added to the bacterial media and incubated for 48 hours at 30°C. Next, the aggregates are immersed inside the growth media for 72 hours at 25°C. After the test period, the aggregates are tested for basic characterization. It is prudent to mention that the biotreatment protocol varies based on the bacteria, culture media, concentration of chemicals, temperature, etc. For example, Wang et al. (2017) reported different chemicals and method for the bio-deposition treatment on RCA than Qiu et al. (2014).

### Carbonation

In this treatment, an airtight compact steel container with a volume of about 100 L is taken as reported by Li et al. (2018). The container is connected with a CO_2_ storage tank and an air pump. Before the treatment, RCA is kept in a dry chamber at 25 ± 3°C with a relative humidity of about 50 ± 5%. First, the chamber is vacuumed to 0.6 bar pressure using the pump and then RCAs are placed inside the chamber. Next, CO_2_ is injected at a constant pressure and finally kept constant at 1 bar for 7 days. After the treatment period, the treated aggregates are tested for basic characterization.

### Cement slurry coating

Martirena et al. (2016) concluded that a layer of cement slurry of water–cement ratio between 0.25 and 0.5 can improve RCA properties. Cement slurry is prepared with a specified water–cement ratio. RCA is mixed with the cement slurry for 5 minutes. After drying for 24 hours, the aggregates are placed in a moist environment for curing.

Major advantages and drawbacks of different treatment methods are shown in Supplemental Table S1. Moreover, the drawbacks of any method indicate a qualitative measure. Thus, an LCA is deemed necessary to get more insights (especially, environmental impacts) of any treatment method on RCA.

## Materials and methods

The guidelines for LCA methodology as provided in ISO14040 and ISO 14044 were adopted for the analysis. LCA tools provide the quantitative and scientific basis of any designed project consisting of the following steps: (1) defining goal and scope of the project, (2) model of the project with all associated inputs and outputs (the basic data collection is known as life cycle inventory (LCI)), (3) environmental impact assessment, and (4) interpretation of the analysis results (ISO 14040, 2006; ISO 14044, 2006; Pré Consultants, 2016). The present LCA was carried out using the SimaPro® 8.1 software with Ecoinvent as the primary database and follows the same guidelines provided in ISO14040 (ISO 14040, 2006) and ISO 14044 (ISO 14044, 2006). The LCA software is scientifically proven to be valid in this type of research and has been used extensively worldwide (Bindra et al., 2015; Mistri et al., 2021).

### Defining the goal and scope

The goal of the study is to find the most suitable treatment method among all published techniques that are available in the literature supporting effective recycling of C&D waste as aggregate in concrete using LCA. The study is based on a particular location and for analysis, Kharagpur, India is chosen as the location of the treatment plant for RCA. Available methods for both removal and strengthening of AM is considered to treat RCA for better performance in terms of water absorption of aggregate. The scope of the study is based on the cradle to gate theory where the gate is defined as the final treated RCA material. The process consists of three segments: C&D waste collection, processing of this waste into aggregate at crushing plant and treatment plant. At the treatment plant, RCA is considered to be treated with different property enhancement approaches. The concrete production using different treated aggregate has excluded from this study. The primary reason behind this is related to the unavailability of data for engineering property of concrete with a single concrete mix and obviously the intended objective.

### System boundary

**Figure 1.**
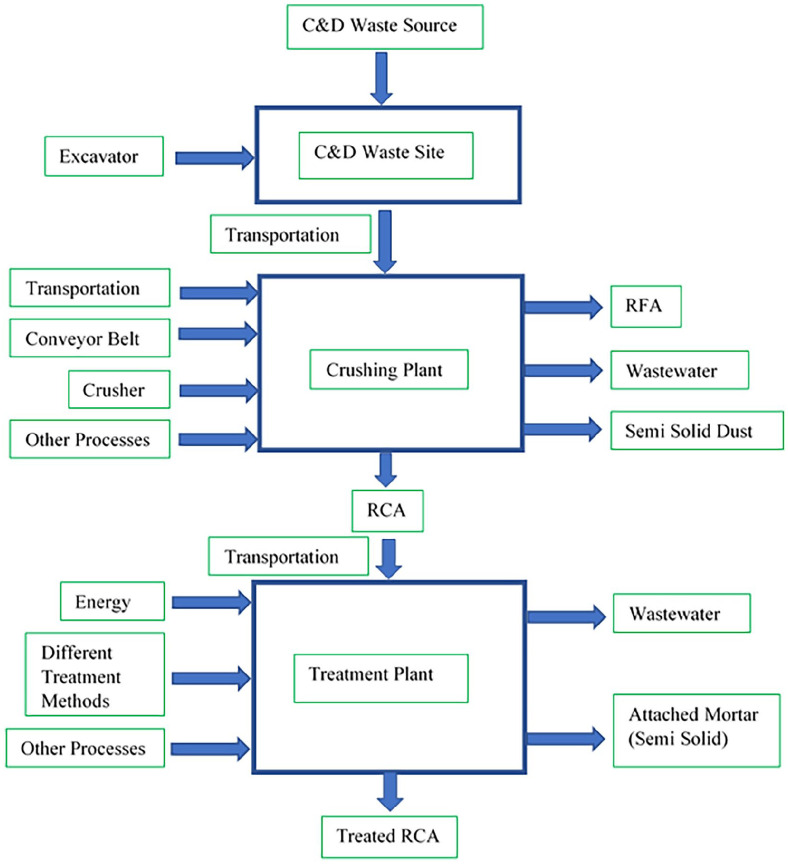
Generalized system boundary for different treated RCA.

The system boundary for this study is limited up to treated aggregate preparation (ref. Figure 1). The system boundary includes the application of different treatment methods on RCA. The collection of raw C&D waste at the site, transportation of waste to the crushing plant, processing of aggregate and transportation of the RCA to the treatment plant are assumed to be the same for all the methods. Among different LCI, only the data for treatment plant vary. Electricity and fuel consumption for operation and maintenance are included, while the construction of buildings and manufacturing of equipment are excluded from the study.

### Functional unit

The primary objective of the study is to find out the most suitable treatment method for RCA with minimum environmental impact. On this basis, the functional unit of the study is considered the preparation of 1 tonne of treated RA, considering reduction in water absorption after any suitable treatment method. Data for water absorption of both control and treated aggregate were collected from the literature. However, it is noteworthy to mention that in many conventional concrete-related LCA study, compressive strength was considered as the functional unit (Ahmed Shaikh et al., 2019; Mistri et al., 2021). This study deals with material level, not in a composite level; therefore, the reason behind choosing water absorption as a functional unit makes justification. The key assumptions of the study are given in Supplemental Material.

### Life cycle inventory

The most important task of conducting LCA is the data collection (Pré Consultants, 2016, 2018). LCI is a list of all input and output database within the previously stated system boundary. LCI consists of the input of raw materials, energy inputs, consumption of fuel and some sort of output as a waste product or secondary products. The raw material for the present analysis is the same source of C&D waste. The C&D waste site is located 25 km from the crushing plant and the treatment plant is located 35 km from the crushing plant.

For fuel, transportation of materials, cement production, water consumptions, chemical used for treatment are acquired from Ecoinvent 3.01 database. These are known as background database (Pradhan et al., 2019; Pré Consultants, 2016). The electricity used in any of the operations within the system boundary was obtained from West Bengal, India energy mix. In West Bengal, thermal, hydro, nuclear and renewable energy systems generate 9220.19, 315.88, 92.88 and 262.71 MWh of electricity per year (Energypedia, 2015).

Emission database was improved by linking these values for the electricity mix in West Bengal to determine the emission factor. For any specific treatment on RCA, the corresponding energy inputs, equipment’s used and other materials were linked with Ecoinvent 3.01 database (https://ecoinvent.org/). The production details for RCA data can be found in Mistri et al. (2021) and Pradhan et al. (2019). The same source of RCA was used for the present analysis at the same laboratory at IIT Kharagpur, India.

### Life cycle impact assessment

Life cycle impact assessment (LCIA) is a measure of different impacts from a predefined LCI. The basic steps for LCIA include (1) selection of the relevant impact categories, (2) classification and characterization of the assigned LCI to the previously selected impact categories, and (3) normalization of impacts (Colangelo et al., 2018; Hossain et al., 2016; Marinković et al., 2010). For the present analysis, the problem-oriented mid-point approach and damage-oriented end-point approach were considered for impact assessment (Hossain et al., 2016; Marinković et al., 2010). The mid-point approach deals with global warming, acidification, ozone layer depletion, eutrophication, photochemical oxidant creation, abiotic depletion and human toxicity. These can be evaluated using CML, ReCiPe, IMPACT 2002+ methods (Pradhan et al., 2019), whereas the end-point approach deals with resources, ecosystem quality, climate change and human health (Ismaeel, 2018; ISO 14040, 2006; ISO 14044, 2006). An impact assessment was estimated using the SimaPro software which is one of the most used LCA software globally which follows ISO (ISO 14040, 2006; ISO 14044, 2006) guidelines. Environmental profiles associated with different removal and strengthening methods were assessed using the LCA software tool SimaPro (Version 8.1). IMPACT 2002+ method was used for impact analysis. The impact assessment was performed for all impact categories. Sensitivity analysis was performed by varying the energy mix to evaluate the impact of the shift from fossil-based to renewable-based energy sources.

### Reduction in water absorption value after treatment

High water absorption is one of the prime drawbacks of using RCA in fresh concrete (Behera et al., 2014). As the present LCA study deals with different treatment methods on RCA, a common property that has already been reported in published literature should be compared. From the literature, water absorption property has found mostly reported. Moreover, from this, the improvement after any suitable treatment can be easily understood. The details of improvement in water absorption after any treatment are shown in Tables 1 and 2. The information in the tables will guide to understand the property enhancement after a specific treatment on RCA.

**Table 1.** Water absorption of RCA after different removal of AM techniques.

Methods	ID	Types/class	WA (before treatment)	WA (after treatment)	% WA reduced
Mechanical grinding (Dimitriou et al., 2018)	RM1	Energy	7.20	3.70	48.61
Autogenous cleaning (Pepe, 2015)	RM2	Class 1	4.94	4.01	18.83
Pre-soaking in acid solution (Tam et al., 2007)	RM3	H_2_SO_4_ (0.1M)	2.04	1.84	10.09
Pre-soaking in acid solution (Tam et al., 2007)	RM4	HCl (0.1M)	2.04	1.79	12.14
Pre-soaking in acid solution (Tam et al., 2007)	RM5	H_3_PO_4_ (0.1M)	2.04	1.88	7.84
Thermal treatment (Pandurangan et al., 2016)	RM6	500°C	4.58	3.80	17.03

AM: attached mortar; RCA: recycled coarse aggregate.

**Table 2.** Water absorption of RCA after different strengthening of AM techniques.

Methods	ID	Types/class	WA (before treatment)	WA (after treatment)	% WA reduced
Polymer impregnation (Kou and Poon, 2010)	SM1	PVA (10%)	6.84	3.16	53.79
Cement slurry coating (Martirena et al., 2016)	SM2	Cement slurry	5.35	3.00	43.93
MICP (Mistri et al., 2021)	SM3	MICP	5.40	1.59	70.56
MICP (Qiu et al., 2014)	SM4	Bacteria	8.90	7.57	15.00
MICP (Wang et al., 2017)	SM5	Bacteria	6.65	5.34	19.69
Accelerated carbonation (Li et al., 2017)	SM6	100% CO_2_	6.20	5.01	19.22

*Note 1*: MICP treatment from different literature was considered under strengthening of AM techniques.

*Note 2*: It can be seen that mechanical grinding (48.61%) and autogenous cleaning (49.25%) show a higher percentage of reduction in water absorption among different removal of AM approaches. On the other hand, MICP (Mistri et al., 2021) treatment (70.56%) and polymer impregnation (53.79%) show a high reduction percentage of water absorption after the treatment on RCA among strengthening of AM approaches.

AM: attached mortar; RCA: recycled coarse aggregate; MICP: microbial-induced calcium carbonate precipitation.

## Results and discussion

LCA results are assessed in two different categories: (1) removal of AM methods and (2) strengthening of AM methods. Moreover, for each segment, both the mid-point and end-point impact assessments are considered. In mid-point impact categories, major contributors are identified (Humbert et al., 2015; Rosado et al., 2017) and discussed separately for both the removal and strengthening cases. In end-point impact categories, a normalization plot is obtained. A comparison of scenarios considering both the mid-point and end-point impact assessments helps to take a suitable decision. Furthermore, a sensitivity analysis based on future renewable energy gives more insights.

### Environmental profiles for removal of AM methods

#### Mid-point impact assessment

**Figure 2.**
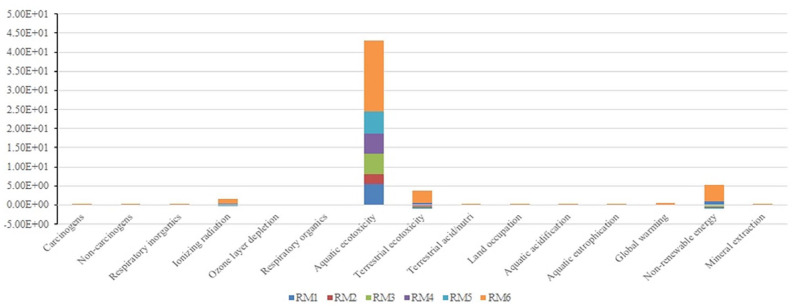
Identification of major impact category for AM removal methods. AM: attached mortar.

It is seen that ionizing radiation, aquatic ecotoxicity, terrestrial ecotoxicity, global warming and non-renewable energy are the major emission contributors (Table 3). Interestingly, these five impact categories (Figure 2) majorly contribute at least one component of damage assessment or end-point characterization factors (i.e. human health, ecosystem quality, climate change and resources). Thus, it is noteworthy to discuss each of the major mid-point contributors separately.

**Figure 3.**
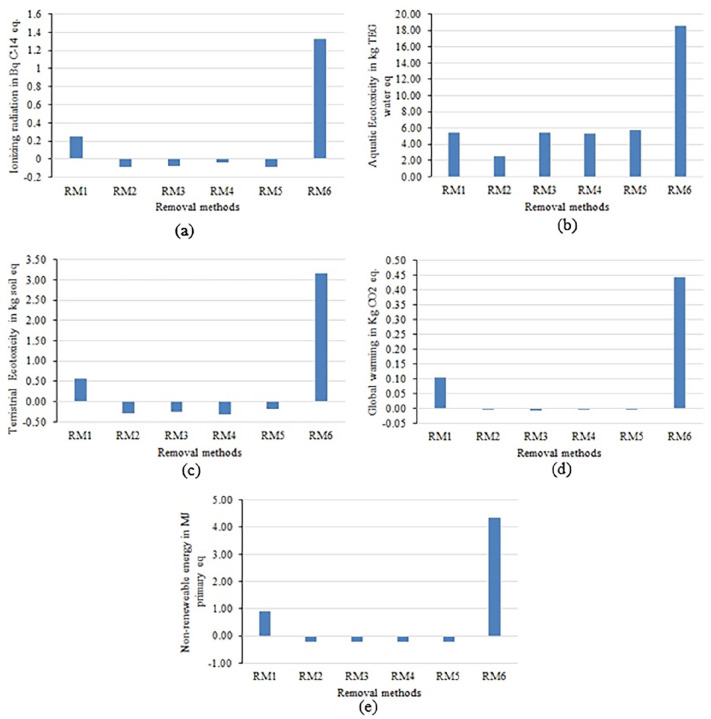
Major mid-point characterization results for AM removal methods: (a) ionizing radiation, (b) aquatic ecotoxicity, (c) terrestrial ecotoxicity, (d) global warming and (e) non-renewable energy. AM: attached mortar.

- **Ionizing radiation:** This characterization factor (CF) is assessed in terms of Bq Carbon-14 (Bq C-14 eq) in air or water (Humbert et al., 2015). It can be seen that RM6 is the major emission contributing method among all removal of AM categories (Figure 3(a)). The value for ionizing radiation emission factor is about 1.33 Bq C-14 eq and which is significantly higher than RM2 (−9.26E−02 Bq C-14 eq). The prime reason behind this is the huge thermal load that is associated with RM6.
- **Aquatic ecotoxicity:** This CF is assessed in terms of kg, triethylene glycol (kg TEG water) into water (Humbert et al., 2015). Again, RM6 is the major emission contributing method among all removal of AM categories (Figure 3(b)). The value for aquatic ecotoxicity emission factor is about 18.5 kg TEG water. Interestingly, RM3, RM4 and RM5 have similar emission contribution for this CF and mainly composed of acid treatment on aggregates. Moreover, RM2 shows the lowest contributor (2.53 kg TEG water).
- **Terrestrial ecotoxicity:** This CF is assessed in terms of kg, triethylene glycol (kg TEG soil) into the soil (Humbert et al., 2015). It can be seen that RM6 is the major emission contributing method among all removal of AM categories (Figure 3(c)). The value for terrestrial ecotoxicity emission factor is about 3.17 kg TEG soil. Moreover, RM4 shows the lowest contributor (−3.25E−01 kg TEG soil). It can be seen that acid treatments (RM3, RM4 and RM5) do not contribute to this CF. Moreover, RM1 contributes 5.54E−01 kg TEG soil to this CF.
- **Global warming:** This CF is assessed in terms of kg CO_2_-eq into the air (Cheela et al., 2021; Humbert et al., 2015). Again, RM6 is the major emission (4.42E−01 kg CO_2_-eq) contributing method among all removal of AM categories (Figure 3(d)). Moreover, RM2 shows the second most contributor (1.03E−01 kg CO_2_-eq). Acid treatments (RM3, RM4 and RM5) do not contribute to the CF much.
- **Non-renewable energy:** This CF is assessed in terms of energy consumption (MJ) (Humbert et al., 2015). RM1 is the second most emission contributor after RM6. The prime reason behind this is related to the energy consumption during the aggregate treatment process (Figure 3(e)). The value for the non-renewable energy emission factor for RM6 is about 4.32 MJ.

In the rest of the impact categories, all the methods contribute significantly less to environmental emissions. Overall, based on the mid-point characterization and contribution towards environmental emissions, the ranking of scenarios is RM6 > RM1 > RM5 > RM4 > RM2 > RM3.

**Table 3.** Mid-point characterization results for AM removal methods.

Impact category	Treatment methods						Unit
	RM1	RM2	RM3	RM4	RM5	RM6	
Carcinogens	7.99E−04	5.58E−04	5.25E−04	5.47E−04	6.34E−04	1.59E−03	kg C_2_H_3_Cl eq
Non-carcinogens	3.40E−04	−2.92E−05	−1.15E−05	−3.23E−05	2.98E−04	1.49E−03	kg C_2_H_3_Cl eq
Respiratory inorganics	2.32E−04	−8.75E−06	−1.42E−05	−1.36E−05	−1.13E−05	9.81E−04	kg PM2.5 eq
Ionizing radiation	2.53E−01	−9.26E−02	−7.76E−02	−3.82E−02	−8.77E−02	1.33E+00	Bq C-14 eq
Ozone layer depletion	−5.81E−10	−1.15E−09	−9.45E−10	−1.27E−10	−8.65E−10	1.11E−09	kg CFC-11 eq
Respiratory organics	−8.67E−06	−1.37E−05	−1.27E−05	−1.34E−05	−1.18E−05	6.34E−06	kg C_2_H_4_ eq
Aquatic ecotoxicity	5.43E+00	2.53E+00	5.47E+00	5.30E+00	5.70E+00	1.85E+01	kg TEG water
Terrestrial ecotoxicity	5.54E−01	−2.93E−01	−2.47E−01	−3.25E−01	−1.88E−01	3.17E+00	kg TEG soil
Terrestrial acid/nutri	1.82E−03	−3.92E−04	−4.09E−04	−4.40E−04	−3.61E−04	8.68E−03	kg SO_2_ eq
Land occupation	−3.96E−04	−1.49E−03	−1.42E−03	−1.64E−03	−7.83E−04	2.91E−03	m2org.arable
Aquatic acidification	7.42E−04	−3.58E−05	−4.39E−05	−4.93E−05	−2.57E−05	3.16E−03	kg SO_2_ eq
Aquatic eutrophication	1.91E−05	−2.72E−07	−3.61E−07	−2.96E−07	5.30E−06	7.95E−05	kg PO_4_ P-lim
Global warming	1.03E−01	−5.67E−03	−6.73E−03	−6.14E−03	−5.22E−03	4.42E−01	kg CO_2_ eq
Non-renewable energy	8.86E−01	−2.29E−01	−2.29E−01	−2.23E−01	−2.10E−01	4.34E+00	MJ primary
Mineral extraction	4.57E−04	−5.41E−04	−3.54E−04	−3.93E−04	5.20E−05	3.55E−03	MJ surplus

*Note*: RM1 = removal of mortar using mechanical grinding method; RM2 = autogenous cleaning method; RM3 = pre-soaking in H_2_SO_4_ acid solution; RM4 = pre-soaking in HCl acid solution; RM5 = pre-soaking in H_3_PO_4_ acid solution; RM6 = thermal treatment method.

AM: attached mortar.

#### End-point impact assessment

**Figure 4.**
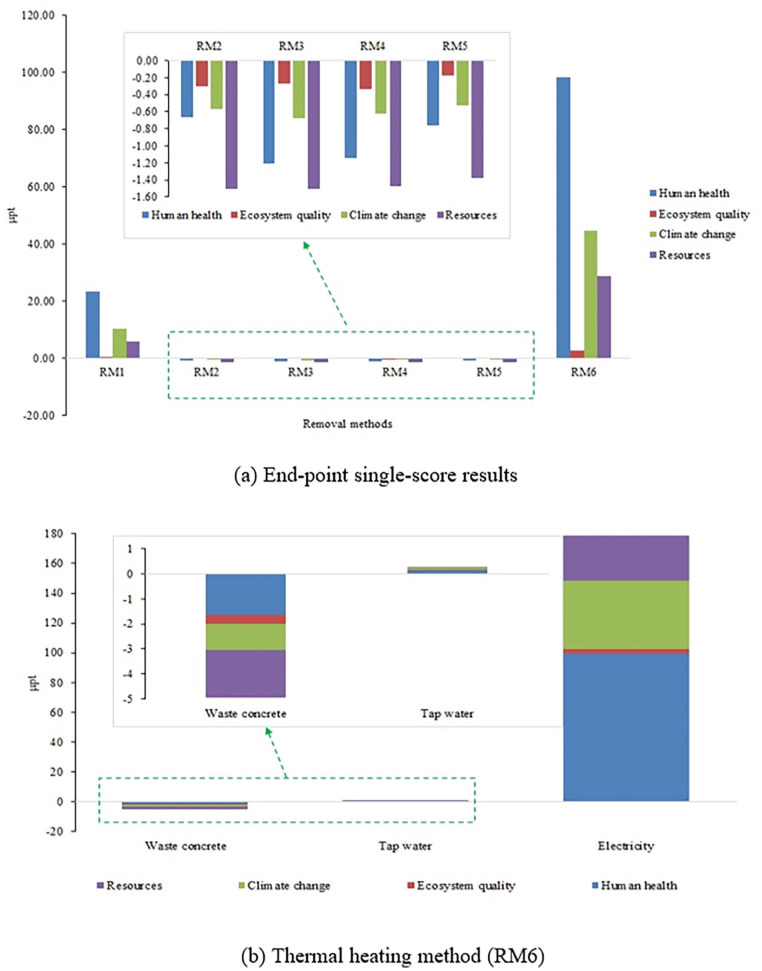
End-point single-score results (a) for AM removal methods and (b) breakdown of thermal heating method (RM6). AM: attached mortar.

The single-score result represents previous steps such as inventory, characterization, normalization and weighting (Cheela et al., 2021; Humbert et al., 2015; Pradhan et al., 2019). Thus, a single-score result can be considered as the final result. However, the prime drawback associated with the single-score method is that the many impacts are out of scope or beyond the calculation (Pré Consultants, 2016). Therefore, individual impact results are also necessary. The utilization of RA is an emission offsetting unit operation. Environmental profiles based on the end-point characterization are presented as the single score for the AM removal methods in Figure 4(a). The thermal treatment (RM6) contributes to the maximum environmental emissions in all the impact categories followed by mechanical grinding (RM1). The removal methods RM2 to RM5 offset the environmental emissions in all the impact categories. In RM6, 56.4% emissions contribute to human health, 1.6% to ecosystem quality, 25.6% to climate change and 16.4% to resources. In RM1, 58.3% emissions contribute to human health, 1.1% to ecosystem quality, 26.1% to climate change and 14.5% to resources. In RM2 to RM5 scenarios, the major emission offsetting impact category is resources with a maximum in RM2 (49.4%) and minimum in RM3 (42.1%). In the human health impact category, the maximum emission offsetting method is RM3 (33.0%) and the minimum is RM2 (21.7%). In the ecosystem quality impact category, the maximum emission offsetting method is RM2 (10.1%) and the minimum is RM5 (6.2%). In the climate change impact category, the maximum emission offsetting method is RM2 (18.8%) and the minimum is RM4 (17.4%). Overall, based on the end-point characterization and contribution towards environmental emissions, the ranking of scenarios is RM6 > RM1 > RM5 > RM2 > RM4 > RM3.

The process-wise single-score values for the RM6 scenario are presented in Figure 4(b). Electricity is the major emission contributing unit operation, while recycling of waste concrete is the emission offsetting process. In the Indian scenario, the fuel source of electricity generation is predominantly coal. The shift towards renewable sources of energy (e.g. hydroelectric power) will reduce the associated environmental emissions in this process.

### Environmental profiles for strengthening of AM methods

#### Mid-point impact assessment

**Figure 5.**
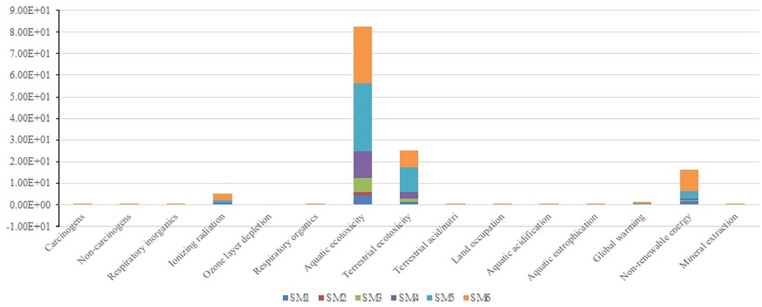
Major impact category for strengthening of AM methods. AM: attached mortar.

Similar to removal of AM, ionizing radiation, aquatic ecotoxicity, terrestrial ecotoxicity, global warming and non-renewable energy are the major emission contributors (Table 4 and Figure 5). Each of the major mid-point contributors is discussed separately.

**Figure 6.**
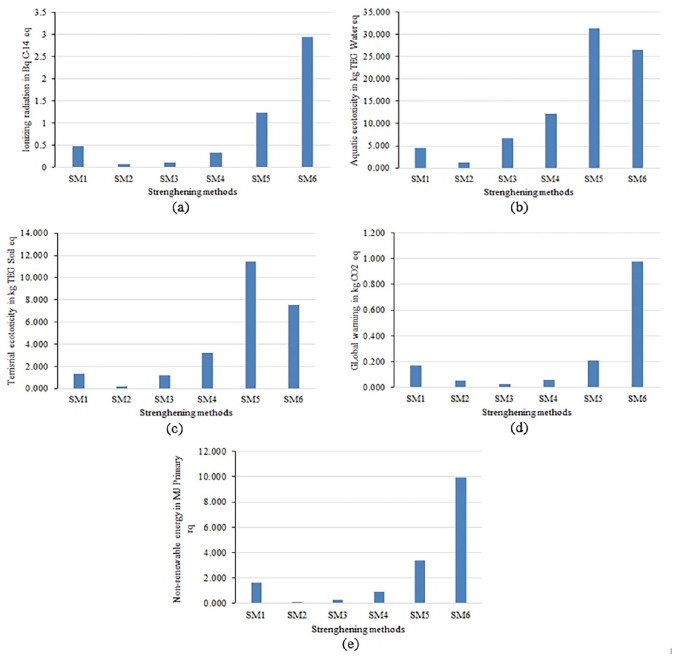
Major mid-point characterization results for strengthening of AM methods: (a) ionizing radiation, (b) aquatic ecotoxicity, (c) terrestrial ecotoxicity, (d) global warming and (e) non-renewable energy. AM: attached mortar.

- **Ionizing radiation:** SM6 is the major emission contributing method among all strengthening of AM categories (Figure 6(a)). The value for ionizing radiation emission factor is about 2.95 Bq C-14 eq and which is significantly higher than SM2 (8.19E−02 Bq C-14 eq) and SM1 (4.82E−01 Bq C-14 eq). The second most contributor for this CF is the SM5 (1.24 Bq C-14 eq).
- **Aquatic ecotoxicity:** Instead of SM6, SM5 is the major emission contributing method among all strengthening of AM categories (Figure 6(b)). The value (SM5) for aquatic ecotoxicity emission factor is about 31.3 kg TEG water. SM6 is the second most emission contribution for this CF and the value is 26.5 kg TEG water. Moreover, SM2 shows the lowest contributor (1.25 kg TEG water).
- **Terrestrial ecotoxicity:** SM5 is the major emission contributing method among all strengthening of AM categories (Figure 6(c)). The value for terrestrial ecotoxicity emission factor is about 11.5 kg TEG soil. Moreover, SM2 (2.10E−01 kg TEG soil) and SM3 (1.19 kg TEG soil) show the lower contributor among all strengthening methods.
- **Global warming:** SM6 is the major emission (9.79E−01 kg CO_2_-eq) contributing method among all strengthening of AM categories (Figure 6(d)). Moreover, SM3 shows the lowest contributor (2.49E−02 kg CO_2_-eq). Although SM5 is the second most contributor but compared to SM6, the impact is significantly lesser.
- **Non-renewable energy:** SM6 is the most emission contributor (9.90 MJ). The prime reason behind this is related to the energy consumption during the accelerated carbonation treatment process (Figure 6(e)). SM2 (9.74E−02 MJ) and SM3 (2.86E−01) result in lower contributors for non-renewable energy emission factors. The energy generation using coal as a fuel source is the contributing substance in these scenarios.

In the rest of the impact categories, all the methods contribute significantly less to environmental emissions. Overall, based on the mid-point characterization and contribution towards environmental emissions, the ranking of scenarios is SM6 > SM5 > SM4 > SM3 > SM1 > SM2.

**Table 4.** Mid-point characterization results for strengthening of AM methods.

Impact category	Treatment methods						Unit
	SM1	SM2	SM3	SM4	SM5	SM6	
Carcinogens	7.29E−04	2.94E−04	−2.12E−04	−1.18E−03	−5.17E−03	3.45E−03	kg C_2_H_3_Cl eq
Non-carcinogens	6.00E−04	5.40E−04	3.09E−04	7.68E−04	2.62E−03	3.51E−03	kg C_2_H_3_Cl eq
Respiratory inorganics	3.72E−04	3.01E−05	2.12E−05	6.14E−05	2.04E−04	2.16E−03	kg PM2.5 eq
Ionizing radiation	4.82E−01	8.19E−02	1.13E−01	3.40E−01	1.24E+00	2.95E+00	Bq C-14 eq
Ozone layer depletion	2.27E−10	−2.66E−11	1.51E−09	4.52E−09	1.64E−08	4.40E−09	kg CFC-11 eq
Respiratory organics	5.36E−08	1.32E−06	1.13E−05	4.36E−05	1.75E−04	3.65E−05	kg C_2_H_4_ eq
Aquatic ecotoxicity	4.52E+00	1.25E+00	6.68E+00	1.23E+01	3.13E+01	2.65E+01	kg TEG water
Terrestrial ecotoxicity	1.31E+00	2.10E−01	1.19E+00	3.22E+00	1.15E+01	7.54E+00	kg TEG soil
Terrestrial acid/nutri	3.20E−03	5.22E−04	2.48E−04	1.46E−03	5.55E−03	1.96E−02	kg SO_2_ eq
Land occupation	7.57E−04	−6.11E−04	6.65E−04	3.37E−03	1.44E−02	8.79E−03	m2org.arable
Aquatic acidification	1.19E−03	1.52E−04	7.41E−05	2.46E−04	8.36E−04	6.97E−03	kg SO_2_ eq
Aquatic eutrophication	3.05E−05	2.93E−06	3.50E−06	7.72E−06	2.46E−05	1.76E−04	kg PO_4_ P-lim
Global warming	1.69E−01	5.22E−02	2.49E−02	6.13E−02	2.09E−01	9.79E−01	kg CO_2_ eq
Non-renewable energy	1.62E+00	9.74E−02	2.86E−01	9.01E−01	3.41E+00	9.90E+00	MJ primary
Mineral extraction	1.39E−03	4.77E−04	1.54E−03	4.28E−03	1.53E−02	9.92E−03	MJ surplus

*Note*: SM1 = strengthening of mortar using polymer impregnation method; SM2 = cement slurry coating treatment; SM3 = MICP suggested by Mistri et al. (2021); SM4 = MICP suggested by Qiu et al. (2014); SM5 = MICP suggested by Wang et al. (2017); SM6 = accelerated carbonation treatment.

AM: attached mortar; MICP: microbial-induced calcium carbonate precipitation.

#### End-point impact assessment

**Figure 7.**
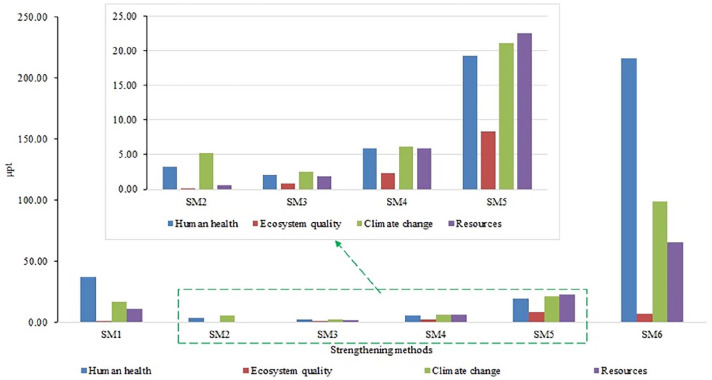
End-point single-score results for strengthening methods.

Environmental profiles based on the end-point characterization are presented as a single score for the AM removal methods in Figure 7. The accelerated carbonation (SM6) contributes to the maximum environmental emissions in all the impact categories followed by MICP3 (SM5) and polymer impregnation (SM1). In SM6, 55.8% of emissions contribute to human health, 1.7% to ecosystem quality, 25.6% to climate change and 16.9% to resources. In SM5, 27.0% emissions contribute to human health, 11.7% to ecosystem quality, 29.7% to climate change and 31.6% to resources. In SM1, 56.4% emissions contribute to human health, 1.6% to ecosystem quality, 25.9% to climate change and 16.1% to resources. In SM2, 35.4% emissions contribute to human health, 1.3% to ecosystem quality, 56.5% to climate change and 6.9% to resources. In SM3, 29.2% emissions contribute to human health, 10.7% to ecosystem quality, 34.3% to climate change and 25.8% to resources. In SM4, 29.1% emissions contribute to human health, 11.7% to ecosystem quality, 29.7% to climate change and 31.6% to resources. In the human health category, SM6 is the major contributing method followed by SM1. In the ecosystem quality category, SM5 is the major contributing method followed by SM4 and SM3. In climate change category, SM5 is the major contributing method followed by SM4. CO_2_ is the major contributing gas in both scenarios. In the resources category, SM5 is the major contributing method followed by SM4.

Overall, based on the end-point characterization and contribution towards environmental emissions, the ranking of scenarios is SM6 > SM5 > SM1 > SM4 > SM2 > SM3. Accelerated carbonation shows significantly higher environmental impacts among the groups. Human health is the main contributor to the same score. Different carbonation treatment needs a separate discussion in detail. In addition to this, MICP treatment followed by Wang et al. (2017) shows a higher environmental impact than MICP by Qiu et al. (2014). The main reason behind this could be the difference in treatment protocol and material used for the treatment. Further research on the same can answer this question. Polymer treatment and MICP by Wang et al. (2017) show almost similar impacts.

### Comparison of scenarios

Comparative ranking based on mid-point, end-point and percentage reduction in water absorption for both removal and strengthening methods is given in Supplemental Table S2. The AM on the RCA surface has a porous structure with high water absorption (Ouyang et al., 2023; Pradhan et al., 2020). In the removal of AM methods, the prime objective is to remove AM from the RCA surface. On the other hand, strengthening of AM enhance the overall properties of RCA by filling the pores, and cracks or forming a barrier on the aggregate surface based on the treatment method adopted. Therefore, before the adoption of treatment methods on RCA for practical application, proper engineering (performance) and environmental considerations should be taken. In removal methods, based on environmental and performance indicators, RM2 is a suggested method. Based on the reduction in water absorption, RM1 (mechanical grinding) is giving the highest reduction. However, based on the environmental indicator, it has the highest impacts associated. Electricity is the major contributing component in this method. Although RM3 (pre-soaking in H_2_SO_4_) is performing best in terms of environmental indicators, performance indicator could be improved using a combination of other removal methods. In strengthening methods, based on environmental and performance indicators, SM3 and SM2 are suggested methods. Based on the reduction in water absorption, SM1 (polymer impregnation) is the second-best performing method. However, based on the end-point environmental indicator, it has the highest impacts associated. In this study, the electricity mix is predominantly coal based (93.21%) and the renewable sources contribute to only 2.65% (Energypedia, 2015). This shift towards the utilization of renewable sources in electricity production (e.g., the electricity mix of Norway), and similar electricity mix can be adopted in this study. Comparative studies based on engineering parameters and long-term performance of concrete using treated aggregate need to be included to understand the application of the aforementioned methods.

### Sensitivity analysis

The Indian energy mix is replaced with Norway electricity mix (98% hydroelectric power based) to estimate the environmental impacts associated with different scenarios in the future. In removal methods based on environmental indicator using the Indian energy mix, RM3 is an environmentally friendly option followed by RM2 and RM4 (Supplemental Table S3). However, in Norway energy mix, RM2 is the best environmentally friendly option followed by RM1 and RM3. In strengthening methods based on environmental indicator using the Indian energy mix, SM3 is an environmentally friendly option followed by SM2 and SM1. However, in Norway energy mix, SM2 is the best environmentally friendly option followed by SM1 and SM6. In the Indian context, the energy mix varies from location to location, based on the energy mix in the site location, the regulatory and administrative authorities can adopt the environmentally sound method. However, engineering properties and long-term performance need to be evaluated for the development of a comprehensive management plan for RCA. The mid-point and end-point characterization results of both the methods are shown in Supplemental Figures S2–S5.

## Conclusion

This paper deals with a comparative LCA on different treatment methods on RCA to find out the most suitable and sustainable method supporting ‘green’ construction. The drawbacks of RCA can be minimized by adopting a suitable treatment method. Among different removal of AM techniques, mechanical grinding (48.61%) and autogenous cleaning (49.25%) show a higher reduction percentage of water absorption value. However, the environmental impact is not the lowest one. From the overall end-point characterization, pre-soaking in acid and autogenous cleaning could be suggested under the removal of AM category. However, the water requirement for the processes is huge and therefore some country with a scarcity of dirking water should avoid this type of removal methods for field practice.

Similarly, among strengthening of AM techniques, MICP (Mistri et al., 2021) treatment (70.56%) and polymer impregnation (53.79%) shows a reduction in water absorption up to a great extent. Now coming to the impact assessment, SM3 and SM2 show the lowest contributor. Therefore, from the overall end-point assessment, MICP (Mistri et al., 2021) and cement slurry coating treatment could be suggested at material level.

From the sensitivity analysis based on Norway energy mix, RM2 is the best environmentally friendly option followed by RM1 and RM3. In strengthening methods based on environmental indicator, SM2 is the best environmentally friendly option followed by SM1 and SM6. Therefore, this study represents the present scenario for India with a focus on the future renewable energy mix. However, the performance of treated aggregate in concrete needs further research. In this context, the concrete performance of MICP-treated RCA shows that method suggested by Mistri et al. (2021) is superior to cement slurry treatment based on environmental impact per MPa of compressive strength of concrete. Therefore, it can be concluded that after assessing material level, a composite-level assessment of different treated RCA needs further investigation before adopting a particular method in an industrial C&D waste recycling project.

## Supplemental Material

sj-docx-1-wmr-10.1177_0734242X231180651 – Supplemental material for Effective method for upcycling construction and demolition waste into concrete: A life cycle approach Supplemental material, sj-docx-1-wmr-10.1177_0734242X231180651 for Effective method for upcycling construction and demolition waste into concrete: A life cycle approach by Abhijit Mistri, Venkata Ravi Sankar Cheela, Brajesh Kumar Dubey, Navdeep Dhami, Sriman Kumar Bhattacharyya, Abhijit Mukherjee and Sudhirkumar V Barai in Waste Management & Research
