# Supplementary material for: Effective method for upcycling construction and demolition waste into concrete: A life cycle approach
Source: Waste Manag Res. 2023 Jun 24;42(3):218–31. doi: 10.1177/0734242X231180651 (PMC10905986; doi:10.1177/0734242X231180651)
Supplement: sj-docx-1-wmr-10.1177_0734242X231180651 – Supplemental material for Effective method for upcycling construction and demolition waste into concrete: A life cycle approach [file sj-docx-1-wmr-10.1177_0734242X231180651.docx]

**Effective method for upcycling construction and demolition waste into concrete: A life cycle approach**

**Supplementary Data**

**Assumptions of the study:** The following summarises key assumptions in scenarios considered for the present study:

1. In different studies, the source of C&D waste is diverse. The C&D waste source may be from a waste recycling plant or RCA can be prepared in the lab after crushing cube or other concrete samples. Assumption of the present LCA calculation considers that the source of RCA is the same for all studies and that is the C&D waste recycling plant. Only treatment methods from different studies were adopted for LCA.
2. The machinery equipment that was used for the treatment of RCA may vary marginally from the actual user. Sometimes, the treatment was applied on a tiny scale and therefore suitable scaling of the machinery was assumed to get 1 tonne of treated RCA.
3. Improvement in water absorption is considered as the functional unit parameter for impact assessment after any treatment at the aggregate level. The performance of concrete using different treated aggregates was not considered under the present scope of analysis.
4. The exact calculation of water consumed in any process is not well-documented in the available literature. Therefore, during the treatment of aggregates (both strengthening of AM and removal of it), consumption of water is considered accordingly. Treated wastewater is used in the aggregate treatment processes and for biocementation, acid soaking treatment-related experiments, deionised water was considered.
5. Assessment of environmental profiles was performed on approaches implemented at site locations across the globe. Geographic location plays a crucial role in the assessment of environmental profiles. For the present LCA, Kharagpur (Kolkata, India) is chosen as the location for all future experiments.
6. The presence of RCA size grade for 20-10mm and 10-4.75 is assumed as 60:40 (Pradhan et al., 2019).
7. Assuming that, for complete immersion processes of 1 tonne of RCA, approximately 1500 L of water is required.

**Tables**

**Table S1.** Major advantages and drawbacks of different property enhancement methods on RCA

| Type of treatment | Methods | Advantages | Drawbacks |
| --- | --- | --- | --- |
| Removal of AM | Mechanical grinding (Dimitriou et al., 2018) | -can remove a significant amount of loose AM  -significant reduction in water absorption | -100% removal of AM is not possible  -requirement of huge mechanical energy |
|  | Autogenous cleaning (Pepe, 2015) | -A small percentage of AM can be separated  -marginal reduction in water absorption | -energy requirement to operate the machinery is high  -not capable of complete removal of AM |
|  | Presoaking in acid solution (Tam et al., 2007) | -capable of removing a small portion of weak AM  -marginal reduction in water absorption | -huge amount of water is required after the treatment  -chemically contaminated water needs suitable recycling |
|  | Thermal treatment (Kumar & Minocha, 2017) | -can remove a significant amount of AM  -considerable reduction in water absorption | -huge thermal energy requirement  -remaining weak AM on the aggregate surface may show very poor performance |
| Strengthening of AM | Polymer impregnation (Kou & Poon, 2010) | -can reduce water absorption of RCA significantly | -this treatment cannot improve the strength of AM  -complex treatment procedure  -operating chamber is a big concern |
|  | Cement slurry coating (Martirena et al., 2016) | -can reduce water absorption as reported.  -has potential of improving strength of RCA | -requirement of additional cement  -this treatment procedure needs further investigation |
|  | Microbial induced carbonate precipitation (MICP) (Qiu et al., 2014) | -can improve RCA internally by filling pores and cracks within AM  -has great potential to improve water absorption as well as weakness of RCA | -the research is in laboratory scale  -different chemicals and a particular type of bacteria are involved in this process. |
|  | Accelerated carbonation (Li et al., 2017) | -can reduce water absorption of RCA by filling of pores with CaCO_3_ | -dealing with 100% CO_2_ is a big task  -there should be sufficient amount of Ca(OH)_2_ within AM of RCA to react with externally applied CO_2_ |

**Table S2.** Environmental offset and comparison of scenario [Indian Energy Mix] (1 and 6 represents the lowest and highest emissions, respectively) along with reduction in water absorption (1= highest reduction and 6= lowest reduction)

| Removal Methods | | | | | | |
| --- | --- | --- | --- | --- | --- | --- |
| Scenario (→) | RM1 | RM2 | RM3 | RM4 | RM5 | RM6 |
| Mid-Point (EI) | 5 | 2 | 1 | 3 | 4 | 6 |
| End Point (EI) | 5 | 3 | 1 | 2 | 4 | 6 |
| Water absorption (PI) | 1 | 2 | 5 | 4 | 6 | 3 |
| Strengthening Methods | | | | | | |
| Scenario (→) | SM1 | SM2 | SM3 | SM4 | SM5 | SM6 |
| Mid-Point (EI) | 2 | 1 | 3 | 4 | 5 | 6 |
| End Point (EI) | 4 | 2 | 1 | 3 | 5 | 6 |
| Water absorption (PI) | 2 | 3 | 1 | 6 | 4 | 5 |
| EI: Environmental Indicator; PI: Performance Indicator | | | | | | |

**Table S3.** Environmental offset and comparison of scenario [Norway Energy Mix] (1 and 6 represents the lowest and highest emissions, respectively)

| Removal Methods | | | | | | |
| --- | --- | --- | --- | --- | --- | --- |
| Scenario (→) | RM1 | RM2 | RM3 | RM4 | RM5 | RM6 |
| Mid-Point (N) | 2 | 1 | 3 | 4 | 6 | 5 |
| End Point (N) | 2 | 1 | 3 | 4 | 6 | 5 |
| Strengthening Methods | | | | | | |
| Scenario (→) | SM1 | SM2 | SM3 | SM4 | SM5 | SM6 |
| Mid-Point (N) | 1 | 2 | 4 | 5 | 6 | 3 |
| End Point (N) | 1 | 3 | 4 | 5 | 6 | 2 |

**Figures**

(iii)

(ii)

(i)

Old ITZ

RCA

Old ITZ

RCA

Cement slurry coating

RCA

New ITZ

Densified zone

**Figure S1.** Visual representation of treatment methods on RCA and corresponding improvement in aggregate properties: (i) untreated RCA, (ii) cement slurry treatment on RCA, (iii) Bio deposition of calcium carbonate (or MICP) treatment on RCA. (Adapted from (Mistri et al., 2023))

[**Note:** In cement slurry treatment, the new layer of cement slurry is outside of the aggregate boundary. Thus, the overall porosity within the attached mortar is almost the same. In MICP, the bacteria fill the pores and densified the attached mortar within RCA, thus overall reduction in water absorption and improvement in mechanical performance can be expected (Mistri et al., 2023).]


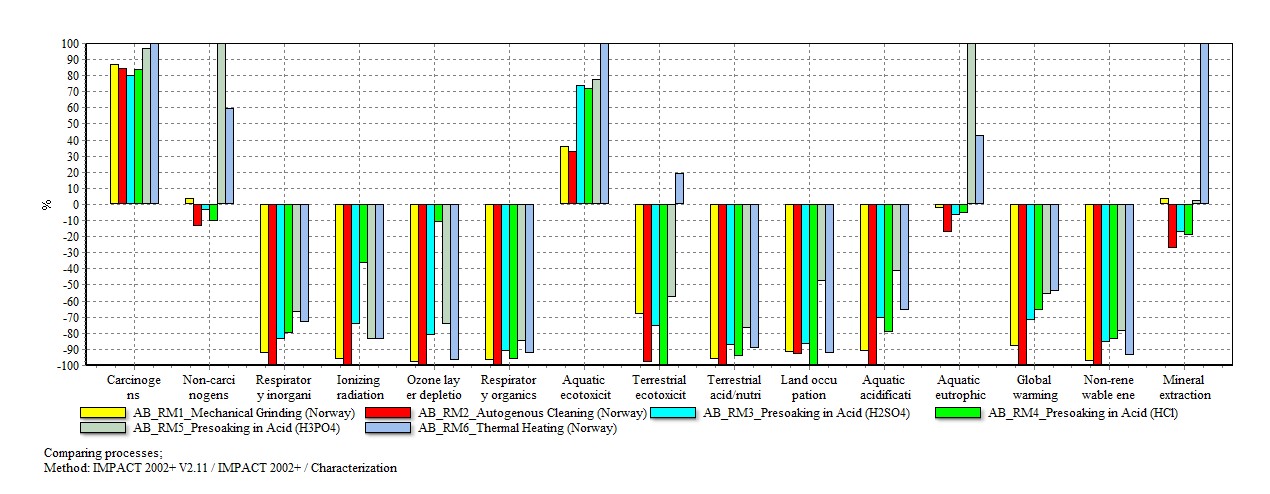


**Figure S2.** Mid-point characterization results for attached mortar removal methods [**Note:** RM1= Removal of mortar using Mechanical grinding method; RM2= Autogenous cleaning method; RM3= Presoaking in H_2_SO_4_ acid solution; RM4= Presoaking in HCl acid solution; RM5=Presoaking in H_3_PO_4_ acid solution; RM6= Thermal treatment method]


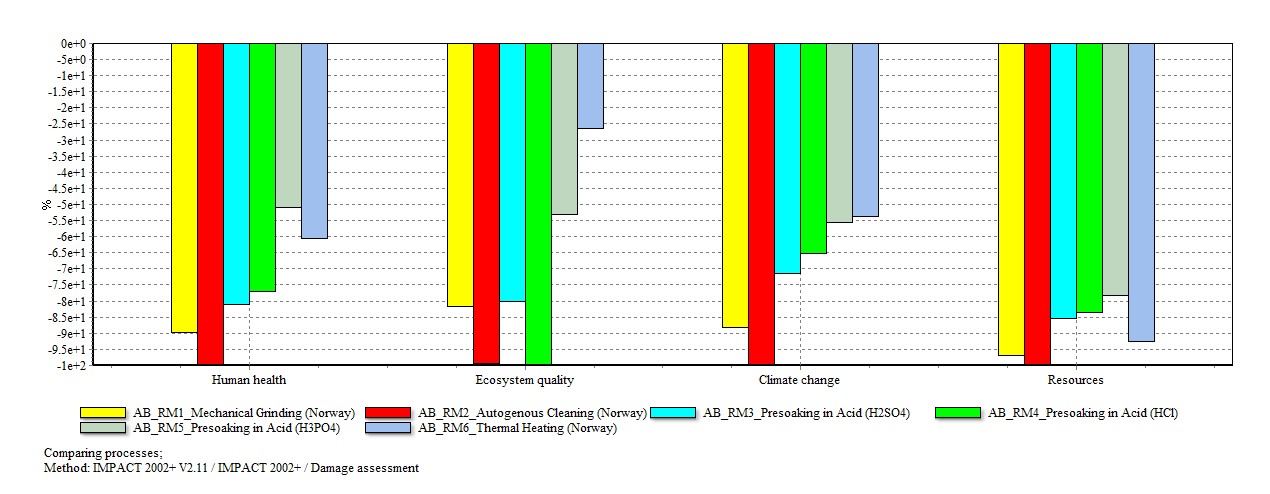


**Figure S3.** End-point Damage Assessment single-score results for attached mortar removal methods


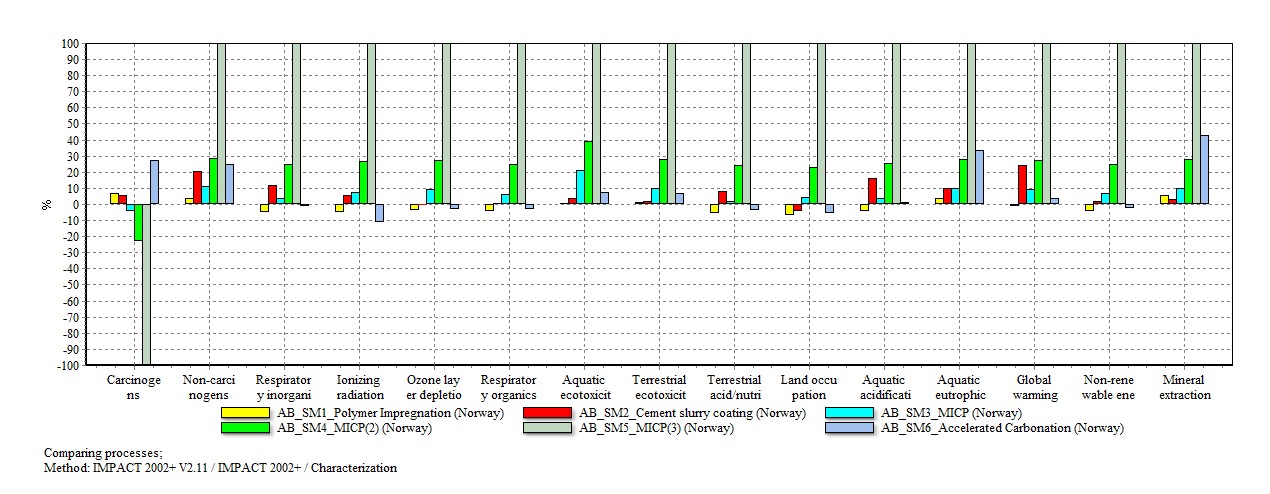


**Figure S4.** Mid-point characterization results for strengthening methods

[**Note:** SM1= Strengthening of mortar using Polymer impregnation method; SM2= Cement slurry coating treatment; SM3= MICP suggested by (Mistri et al., 2021); SM4=MICP suggested by (Qiu et al., 2014); SM5= MICP suggested by (Wang et al., 2017); SM6= Accelerated carbonation treatment]


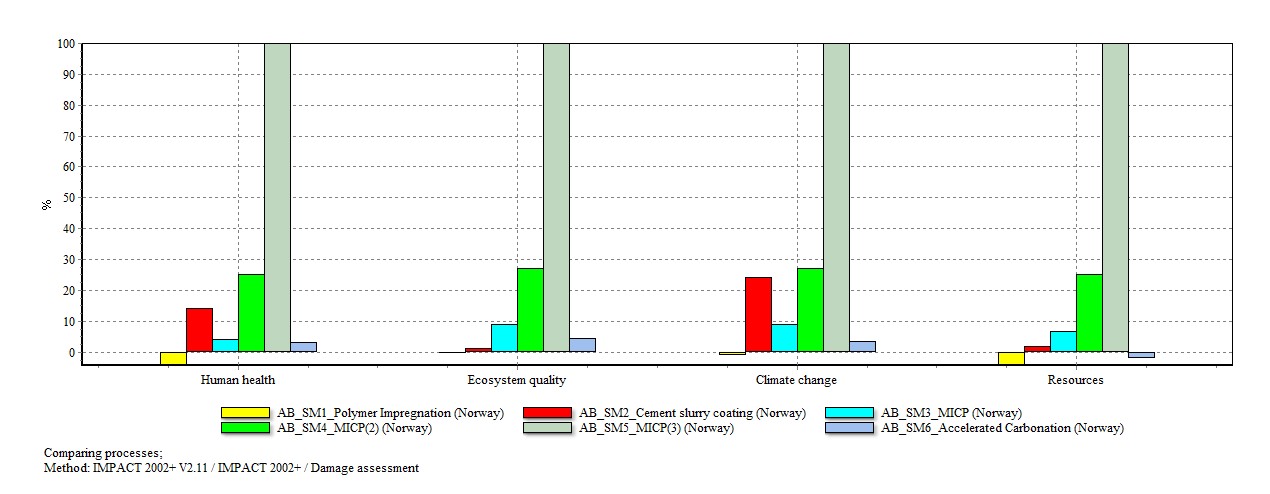


**Figure S5.** End-point Damage Assessment single-score results for strengthening methods

**Reference**

Dimitriou, G., Savva, P., & Petrou, M. F. (2018). Enhancing mechanical and durability properties of recycled aggregate concrete. *Construction and Building Materials*, *158*, 228–235. https://doi.org/10.1016/j.conbuildmat.2017.09.137

Kou, S. C., & Poon, C. S. (2010). Properties of concrete prepared with PVA-impregnated recycled concrete aggregates. *Cement and Concrete Composites*, *32*(8), 649–654. https://doi.org/10.1016/j.cemconcomp.2010.05.003

Kumar, G. S., & Minocha, A. K. (2017). Studies on thermo-chemical treatment of recycled concrete fine aggregates for use in concrete. *J Mater Cycles Waste Manag*, *0123456789*. https://doi.org/10.1007/s10163-017-0604-6

Li, L., Sun, C., Xiao, J., & Xuan, D. (2017). Effect of carbonated recycled coarse aggregate on the dynamic compressive behavior of recycled aggregate concrete. *Construction and Building Materials*, *151*, 52–62. https://doi.org/10.1016/j.conbuildmat.2017.06.043

Martirena, F., Castaño, T., Alujas, A., Orozco-morales, R., Martinez, L., & Linsel, S. (2016). Improving quality of coarse recycled aggregates through cement coating. *Journal of Sustainable Cement-Based Materials*, *0373*(September). https://doi.org/10.1080/21650373.2016.1234983

Mistri, A., Dhami, N., Bhattacharyya, S. K., Barai, S. V., & Mukherjee, A. (2023). Performance of biocement treatment in improving the interfacial properties of recycled aggregate concrete. *Construction and Building Materials*, *369*, 130509. https://doi.org/10.1016/j.conbuildmat.2023.130509

Mistri, A., Dhami, N., Bhattacharyya, S. K., Barai, S. V., Mukherjee, A., & Biswas, W. K. (2021). Environmental implications of the use of bio-cement treated recycled aggregate in concrete. *Resources, Conservation and Recycling*, *167*(July 2020), 105436. https://doi.org/10.1016/j.resconrec.2021.105436

Pepe, M. (2015). *A Conceptual Model for Designing Recycled Aggregate Concrete for Structural Applications*. Springer Theses, Springer International Publishing Switzerland. https://doi.org/10.1007/978-3-319-26473-8

Pradhan, S., Tiwari, B. R., Kumar, S., & Barai, S. v. (2019). Comparative LCA of recycled and natural aggregate concrete using Particle Packing Method and conventional method of design mix. *Journal of Cleaner Production*, *228*, 679–691. https://doi.org/10.1016/j.jclepro.2019.04.328

Qiu, J., Tng, D. Q. S., & Yang, E.-H. (2014). Surface treatment of recycled concrete aggregates through microbial carbonate precipitation. *Construction and Building Materials*, *57*, 144–150. https://doi.org/10.1016/j.conbuildmat.2014.01.085

Tam, V. W. Y., Tam, C. M., & Le, K. N. (2007). Removal of cement mortar remains from recycled aggregate using pre-soaking approaches. *Resources, Conservation and Recycling*, *50*(1), 82–101. https://doi.org/10.1016/j.resconrec.2006.05.012

Wang, J., Vandevyvere, B., Vanhessche, S., Schoon, J., Boon, N., & de Belie, N. (2017). Microbial carbonate precipitation for the improvement of quality of recycled aggregates. *Journal of Cleaner Production*, *156*, 355–366. https://doi.org/10.1016/j.jclepro.2017.04.051
